# Tuberculosis Septic Arthritis of the Elbow: A Case Report and Literature Review

**DOI:** 10.7759/cureus.13765

**Published:** 2021-03-08

**Authors:** Pasin Tangadulrat, Sitthiphong Suwannaphisit

**Affiliations:** 1 Department of Orthopedics, Prince of Songkhla University, Songkhla, THA

**Keywords:** tb elbow, tb osteomyelitis, tb septic arthritis, musculoskeletal tuberculosis

## Abstract

Tuberculosis (TB) infections of the musculoskeletal system are rare. A 77-year-old female with chronic left elbow pain for five months was treated by irrigation and debridement of the elbow for a presumed diagnosis of septic arthritis. Her pain and wound condition did not improve, and she was referred to our institution. Plain radiograph and magnetic resonance imaging (MRI) revealed an osteolytic lesion with joint effusion and severe destruction of the elbow joint. We suspected an atypical infection of the elbow due to the chronicity, negative culture results and severe osteoarticular destruction. An open arthrotomy with irrigation and debridement was performed, and the joint was stabilized with a pin and immobilized. A tissue acid-fast bacillus (AFB) stain was positive and *Mycobacterium tuberculosis* culture and polymerase chain reaction (PCR) were also positive. Anti-TB drugs were started for a planned 12-month course, but she developed an adverse drug reaction from the standard regimen and had to be switched to a second-line regimen. The stitches were removed at two weeks and the wound eventually healed. The elbow was immobilized in a posterior slab for six weeks then the pin was removed. At the last follow-up visit seven months after the initial surgery, she had improved, with only mild pain on elbow motion. Her range of motion was 110 degrees of flexion and extension lag of 30 degrees.

TB of the elbow is a rare condition. The presentation is insidious and varies, and can be confused with other elbow conditions. Delayed diagnosis can lead to severe joint destruction and poor outcome. The physician should always suspect a TB elbow in cases of chronic elbow pain with synovitis, especially in areas endemic for TB. Joint fluid aspiration and MRI are the most reliable investigations for diagnosis. Anti-TB drugs are the mainstay of treatment. Appropriate surgical interventions such as drainage, synovectomy and reconstructive procedures will often be required. Collaboration between the orthopedist and an infectious specialist is essential for optimal treatment planning.

## Introduction

Septic arthritis is one of the most common musculoskeletal infections requiring orthopedic care. This condition can cause severe morbidity. The most common organism associated with septic arthritis is *Staphylococcus aureus *[1]. However, various other less common organisms can also lead to these conditions, including *Mycobacterium tuberculosis *(MTb), which can cause chronic septic arthritis.

Tuberculosis (TB) infection of the elbow is rare. The diagnosis is often missed or delayed, and the treatment algorithm is not well established. Herein, we present a case of TB septic arthritis associated with osteomyelitis of the elbow with a literature review and proposed treatment algorithm.

## Case presentation

We report the case of a 77-year-old female with left elbow pain for five months without any prior trauma who received initial treatment at a local hospital in Southern Thailand. She was diagnosed with septic elbow, and open arthrotomy of the elbow was performed and she was given IV antibiotics. The culture results did not identify any specific causative organism at that time. Her symptoms did not resolve, she had a chronic wound and persistent elbow pain, and she was referred to our institution, the major tertiary care and referral center in Southern Thailand, for further investigations and management.

**Figure 1:**
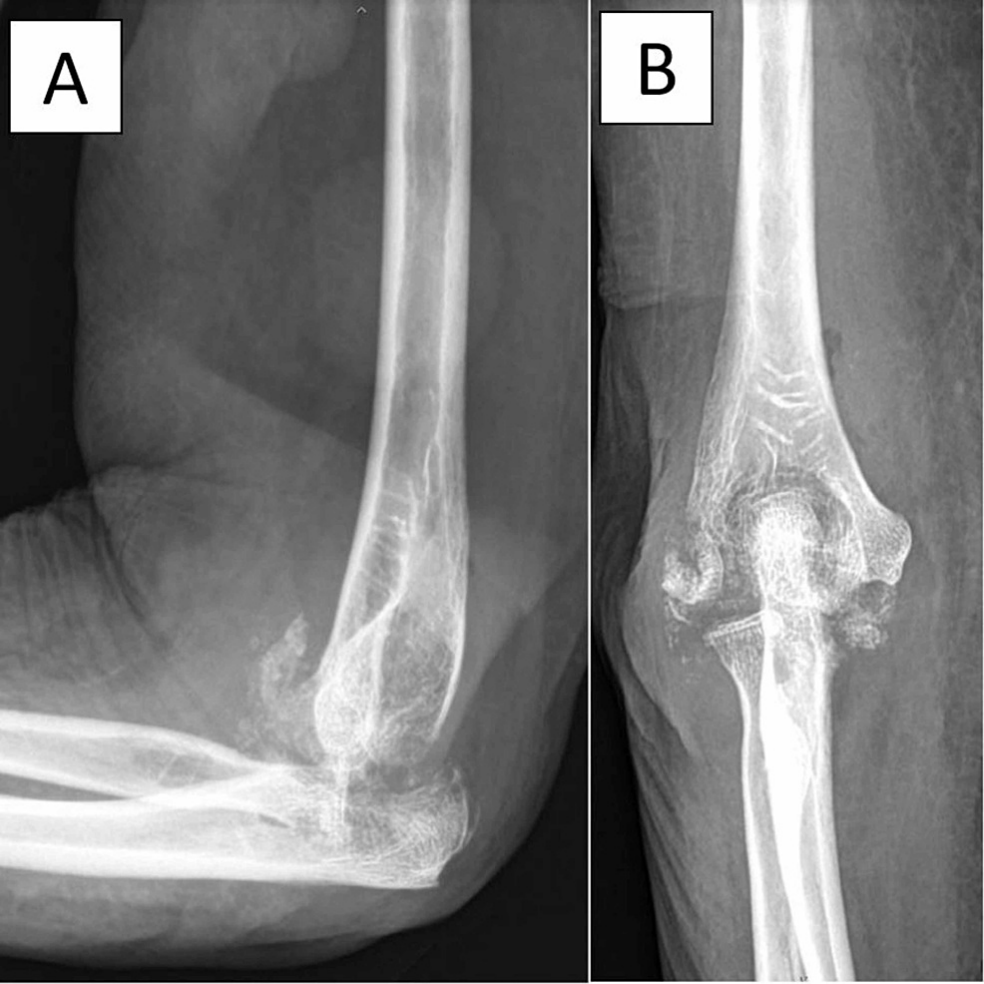
Plain film of the patient’s left elbow showing osteolytic lesions around the elbow joint and severe bone destruction with dislocation of the elbow joint.

She did not have any underlying diseases. The history regarding past pulmonary infection was negative. She did not take any immunosuppressant or herbal medicine. The physical examination at our institute showed a chronic wound at the lateral aspect of the elbow about 0.5 x 2.0 cm in size with clear yellow fluid discharge. The elbow was swollen and warm. Her elbow range of motion was limited due to pain. Laboratory investigations showed a white blood cell count of 4,190/μL with 63% polymorphonuclear neutrophils (PMN). The renal function tests and electrolytes were all within normal limits. Erythrocyte sedimentation rate and C-reactive protein were elevated at 114 mm/hr and 8.48 mg/L, respectively. Radiographs revealed osteolytic lesions around the elbow joint and severe bone destruction with dislocation of the elbow joint (Figures 1A, 1B).

**Figure 2:**
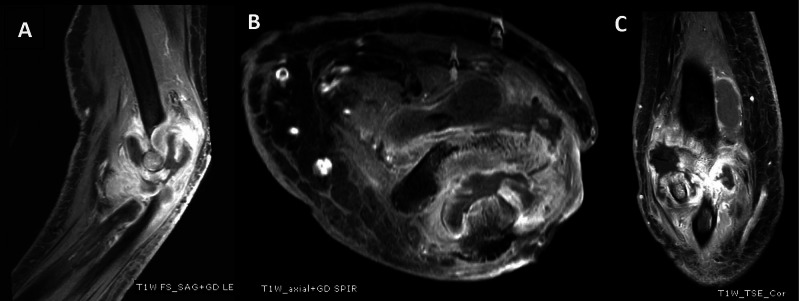
MRI of the left elbow. (A) Sagittal plane T1 with gadolinium. (B) Axial plane T1 with gadolinium. (C) Coronal plane T1 with gadolinium, all showing joint fluid with abnormal bone marrow enhancement and bone destruction. MRI - magnetic resonance imaging

A magnetic resonance imaging (MRI) showed abnormal bone marrow edema involving the distal humerus, proximal radius and ulna with a moderate amount of joint fluid with diffusely enhanced thickened synovium. There was severe destruction of the elbow joint (Figures 2A-2C), which we diagnosed as septic arthritis and osteomyelitis of the elbow.

Due to the chronic presentation, negative culture results and severe destruction of the joint, we suspected an atypical infection such as TB. We suggested surgery for the definitive diagnosis and as having the best likely outcome, and the patient agreed. Chest radiograph was also reviewed and did not find any evidence of pulmonary TB.

In the OR, the elbow was approached posteriorly through a midline longitudinal incision, and necrotic tissue with a cheese-like appearance with no frank pus was found. Copious irrigation and debridement were done.

**Figure 3:**
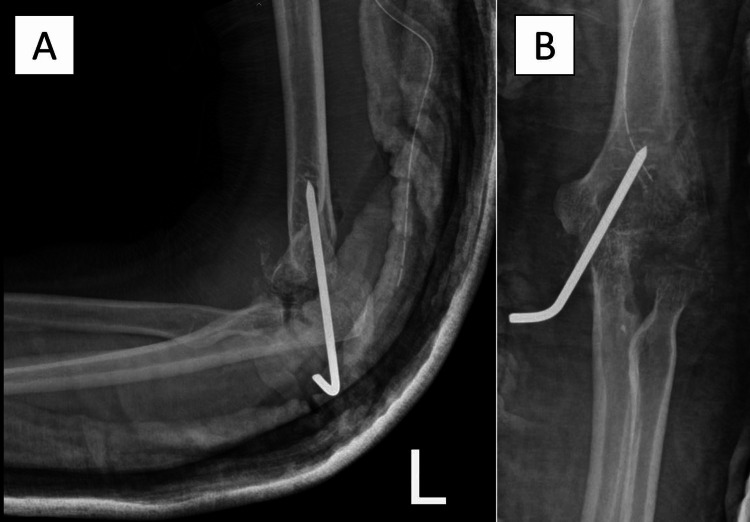
“Hanging elbow procedure” the elbow was stabilized with a Steinmann pin.

A subsequent tissue acid-fast bacillus (AFB) stain was positive with 4 AFB cells/hpf. Polymerase chain reaction (PCR) was also positive for *M. tuberculosis,* and culture later was positive. Two days later, the patient was brought to the operating room for a second time to repeat the irrigation and debridement of the elbow joint because of persistent fever. The ulnohumeral joint was found to be dislocated and was reduced and stabilized with a pin (Figures 3A, 3B). The final diagnosis of TB septic arthritis and osteomyelitis of the elbow was confirmed.

After the operation, a regimen of anti-TB drugs was prescribed by an infectious disease specialist, consisting of isoniazid (INH), rifampin (RIF), ethambutol (EMB) and pyrazinamide (PZA) at dosages of 300, 600, 1,000 and 1,000 mg/day, respectively. The regimen was planned for 12 months according to CDC guidelines [2]. However, early in the treatment, she developed nausea/vomiting with a transaminitis liver profile. Her aspartate aminotransferase (AST) elevated from 21 U/L to 83 U/L and alanine aminotransferase (ALT) elevated from 9 U/L to 31 U/L. The pyrazinamide was considered to be the most likely cause and was changed to levofloxacin 500 mg/day. The liver function test and the symptoms still did not improve, so the regimen was changed to levofloxacin 500 mg/day, streptomycin 600 mg IM three times/week and ethambutol 800 mg/day, following which her symptoms and liver function gradually improved.

**Figure 4:**
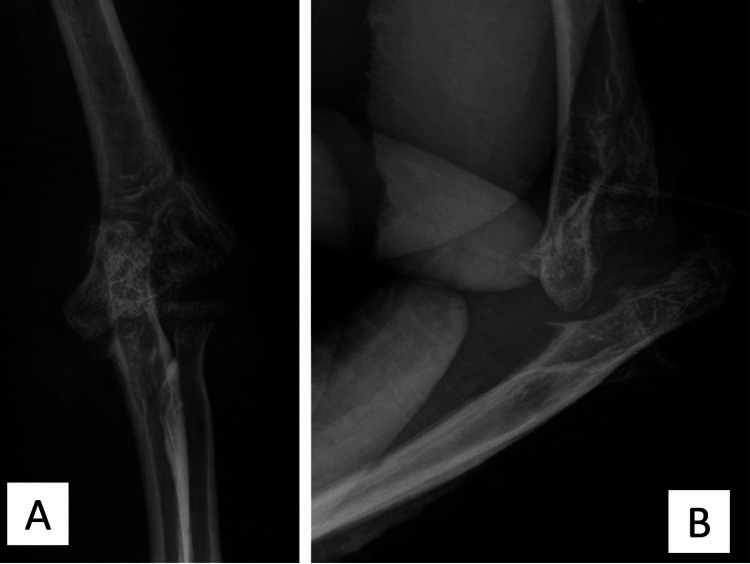
The radiograph shows pseudarthrosis of the elbow.

The wound eventually healed, and the stitches were removed at two weeks' post-operation. The elbow was immobilized in a posterior slab for six weeks, at which time the pin was removed, and the slab replaced with an elbow splint, which allowed a limited extension of 30 degrees. She began a range of motion exercises at eight weeks. At three months' post-operation, she was able to flex her elbow to 110 degrees with an extension lag of 30 degrees. The disabilities of the arm, shoulder and hand (DASH) score improved from 55.8 at presentation to 28.3. At her last follow-up seven months' post-operation, she had mild pain (pain Visual Analog Score = 2-3) with elbow motion with the same arc of motion as at the previous visit. The radiograph shows pseudarthrosis of the elbow (Figures 4A, 4B). After that, she did not come for any more follow-up visits.

## Discussion

Globally, an estimated 10.0 million people were infected with TB in 2018, with the South-East Asia region being especially hard to hit [3]. Extrapulmonary TB (EPTB) is an uncommon form of TB infection. A recent multi-center study from China found that more than half of all EPTB cases had occurred concurrently with pulmonary TB (PTB). Isolated EPTB occurs in about 1/3 of all TB patients [4]. In EPTB patients, the most common presentations are TB lymphadenitis of the neck (7.24%), TB meningitis (7.23%), TB peritonitis (4.79%) and vertebral TB (3.46%). TB in other sites of the musculoskeletal system is rare with a combined incidence of less than 1% [4].

The presentation of ETPB involving the musculoskeletal system varies. TB vertebral osteomyelitis may present with prolonged nonspecific back pain with or without neurological deficit. In late-presenting cases, a kyphosis deformity might be the only presenting symptom [5]. TB septic arthritis of a joint may present as subacute to chronic joint pain and swelling. If the diagnosis is delayed, the disease can progress causing severe cartilage destruction and joint subluxation or dislocation [6].

The pathophysiology of osteochondral TB is well established. First, the MTb infects the macrophages and becomes intracellular. Once inside the cells, Mtb causes a cellular reaction which affects the microenvironment by changing the cellular pH and producing urease. It is also known to produce other enzymes to protect itself from the host phagocytic activity. Cellular immunity cytokines such as TNF-alpha, interferon-gamma and interleukin-12 play essential roles in controlling MTb infection in this stage [7]. 

The treatment of TB infections is mainly either medical treatment or surgical treatment. In the case of musculoskeletal TB, surgical treatment varies by the site and severity of the infection. Common forms of musculoskeletal TB infection such as vertebral TB have well-established surgical indications. In our institution, we normally perform irrigation and debridement and decompression of the neural canal when there is a neurological deficit. Deformity correction and stabilization of the spine is also indicated when there is instability or severe deformity [8]. TB of a hip or knee joint which leads to secondary osteoarthritis will often need joint replacement surgery to alleviate the patient’s symptoms [9].

TB of the elbow joint is extremely rare and there is a paucity of case reports, which are summarized in Table 1.

**Table 1:** Summary of TB elbow case reports ROM - range of motion

Author(s)	Year	Patient characteristics	Diagnosis	Treatment	Outcome
Liao et al. [10]	2017	85-year-old female	TB elbow septic arthritis and osteomyelitis	Anti-TB drugs for 12 months	Little improvement in ROM, persistent pain and wound discharge
Yazıcı et al. [11]	2016	57-year-old male	TB elbow septic arthritis	Anti-TB drugs (duration not stated), Drainage	Decrease pain, limited elbow motion
Rahman et al. [12]	2015	37-year-old male	TB elbow septic arthritis	Anti-TB drugs (duration not stated)	Not reported
Novatnack et al. [13]	2015	69-year-old male	TB elbow septic arthritis	Not reported	Not reported
Jung et al. [14]	2010	50-year-old female	TB elbow septic arthritis and osteomyelitis	Irrigation, anti-TB drugs (duration not stated)	Decrease pain, ROM not reported

A case series by Chen et al. [15] reported the mid- to long-term outcomes of 23 patients diagnosed with TB arthritis of the elbow. Most of the patients presented with chronic elbow pain lasting more than six months and were treated by synovectomy, intra-articular debridement, and curettage. Anti-Tb drugs were given for at least 12 months. The results were quite acceptable, most wounds healed uneventfully, but decreased ROM and residual pain were common. The decreased ROM seemed to be correlated with the severity of osteoarticular destruction and lack of extensive post-op rehabilitation protocol.

Another series done in 1980 by Martini et al. [16] reported the outcomes of conservatively treated TB elbow. All patients were given anti-TB drugs for 12 months, immobilization with a plaster splint for one to two months and proceeding to rehabilitation. Most of them had decreased range of motion, especially in supination and pronation.

From previous evidence, we know that generally, the outcome of TB elbow is quite poor. We believe that there are two major factors that lead to poor results in these cases. First, the patient normally presents late due to the insidious onset nature of this type of TB infection and also there is a high rate of initially missing the correct diagnosis. When diagnosed in the late stage, destruction of the osteoarticular component is severe and even though there are many options for reconstruction, they all usually have a poor outcome. Second, TB infections require specific anti-TB treatments, which are usually prolonged, and thus poor adherence to the anti-TB drugs protocol as well as various side effects from the drugs may hinder complete eradication of the disease.

Aside from the anti-TB drugs, many strategies, both surgical and non-surgical, have been attempted and reported, depending largely on the presence of septic arthritis, osteomyelitis and severity of joint destruction. For elbow infections that present primarily with pain, limited motion or chronic synovitis without significant amounts of collection or associated osteomyelitis, non-surgical treatments with immobilization seem to provide acceptable outcomes. However, when a significant collection is present, drainage and synovectomy are recommended. If the joint is damaged, some form of reconstructive or salvage procedure should be considered. In our case, since the joint was severely damaged, we decided to do a “hanging elbow” procedure consisting of adequate irrigation and debridement and stabilization of the joint by a large pin.

To achieve better outcomes for TB of the elbow, early diagnosis and management are critical. We suggest that in cases of chronic elbow pain with synovitis, TB of the elbow should be included in the initial differential diagnosis. The imaging modality of choice is an MRI that can show features of synovitis, collection and the presence of osteomyelitis. The findings that differentiate TB elbow from other diseases in both MRI and plain film are the presence of severe osteoarticular destruction despite no to mild systemic symptoms. Joint aspiration should be done and sent for AFB staining, PCR and TB culture. Tissue biopsy is another option for early disease with no immediate indication for open irrigation and debridement. Early anti-TB drugs and appropriate surgical intervention will normally improve the outcome. Collaboration with an infectious disease specialist for appropriate anti-TB drugs adjustment if required is essential. In the initial stages of treatment, the patient should be closely monitored for potential side effects from the anti-TB drugs.

## Conclusions

TB infection of the elbow is rare. The presentation is insidious, varies and can be confused with other elbow conditions. Delayed diagnosis can lead to severe joint destruction and poor outcome. The physician should always suspect a TB elbow in cases of chronic elbow pain with synovitis, especially in TB-endemic areas. Joint fluid aspiration and MRI are the best options for diagnosis. Anti-TB drugs are the mainstay for treatment. Appropriate surgical interventions, such as drainage of collection, synovectomy and reconstructive procedures, can be considered necessary. Collaboration between the orthopedist and an infectious specialist is essential for optimal treatment planning.

## References

[1] Urish KL, Cassat JE (2020). Staphylococcus aureus osteomyelitis: bone, bugs, and surgery. Infect Immun.

[2] (2020). Treatment for TB disease. https://www.cdc.gov/tb/topic/treatment/tbdisease.htm.

[3] (2020). Organisation mondiale de la santé. Global tuberculosis report 2019. https://apps.who.int/iris/bitstream/handle/10665/329368/9789241565714-eng.pdf.

[4] Kang W, Yu J, Du J (2020). The epidemiology of extrapulmonary tuberculosis in China: a large-scale multi-center observational study. PloS One.

[5] Ali A, Musbahi O, White VLC, Montgomery AS (2019). Spinal tuberculosis: a literature review. JBJS Rev.

[6] Rodriguez-Takeuchi SY, Renjifo ME, Medina FJ (2019). Extrapulmonary tuberculosis: pathophysiology and imaging findings. Radiogr Rev Publ Radiol Soc N Am Inc.

[7] Hogan JI, Hurtado RM, Nelson SB (2017). Mycobacterial musculoskeletal infections. Infect Dis Clin North Am.

[8] Garfin SR (2018). Rothman-Simeone and Herkowitz’s The Spine Seventh Edition.

[9] Dojode CMR, Joseph G, Shah NN (2018). A deceptive presentation of tuberculosis hip as staphylococcal infection, its successful management and literature review. BMJ Case Rep.

[10] Liao Q, Shepherd JG, Hasnie S (2017). Mycobacterium tuberculosis of the elbow joint. BMJ Case Rep.

[11] Yazıcı A, Kayan G, Yaylacı S (2016). Tuberculous arthritis of the elbow joint: a case report. Eur J Rheumatol.

[12] Rahman J, Patel A, Lam F (2016). Primary tuberculosis of the elbow joint: a case report. Musculoskeletal Care.

[13] Novatnack ES, Protzman NM, Kannangara S, Busch MF (2015). Elbow mycobacterium tuberculosis in America. J Glob Infect Dis.

[14] Jung SS, Lee MK, Lim SH, Kwon Y-M, Choi S-S (2010). Elbow pain proven to be tuberculous arthritis: a case report. Korean J Anesthesiol.

[15] Chen WS, Wang CJ, Eng HL (1997). Tuberculous arthritis of the elbow. Int Orthop.

[16] Martini M, Gottesman H (1980). Results of conservative treatment in tuberculosis of the elbow. Int Orthop.

